# Accelerated Deconvolved Imaging Algorithm for 2D Multibeam Synthetic Aperture Sonar

**DOI:** 10.3390/s22166016

**Published:** 2022-08-12

**Authors:** Bo Wei, Chuanlin He, Siyu Xing, Yi Zheng

**Affiliations:** 1 Institute of Oceanographic Instrumentation, Qilu University of Technology (Shandong Academy of Sciences), Qingdao 266061, China; 2 School of Ocean Technology Sciences, Qilu University of Technology (Shandong Academy of Sciences), Qingdao 266061, China

**Keywords:** multibeam echo sounder, multibeam synthetic aperture sonar, accelerated richardson-lucy iteration, deconvolved beamforming

## Abstract

High-accuracy level underwater acoustical surveying plays an important role in ocean engineering applications, such as subaqueous tunnel construction, oil and gas exploration, and resources prospecting. This novel imaging method is eager to break through the existing theory to achieve a higher accuracy level of surveying. Multibeam Synthetic Aperture Sonar (MBSAS) is a kind of underwater acoustical imaging theory that can achieve 3D high-resolution detecting and overcome the disadvantages of traditional imaging methods, such as Multibeam Echo Sounder (MBES) and Synthetic Aperture Sonar (SAS). However, the resolution in the across-track direction inevitably decreases with increasing range, limited by the beamwidth of the transducer array of MBES. Furthermore, the sidelobe problem is also a significant interference of imaging sonar that introduces image noise and false peaks, which reduces the accuracy of the underwater images. Therefore, we proposed an accelerated deconvolved MBSAS beamforming method that introduces exponential acceleration and vector extrapolation to improve the convergence velocity of the classical Richardson-Lucy (R-L) iteration. The method proposed achieves a narrow beamwidth with a high sidelobe ratio in a few iterations. It can be applied to actual engineering applications, which breaks through the limitation of the actual transducer array scale. Simulations, tank, and field experiments also demonstrate the feasibility and advantages of the method proposed. 3D high-accuracy level underwater acoustical surveying can be achieved through this 2D MBES transducer array system, which can be widely promoted in the field of underwater acoustical remote sensing.

## 1. Introduction

High-resolution underwater acoustical imaging has gradually become a research focus in the fields of underwater resources prospecting and ocean surveying. Many technical branches of underwater acoustical imaging have been developed and widely employed by researchers and sonar manufacturers in the past decades, such as Side Scan Sonar (SSS), Multibeam Echo Sounder (MBES), and Synthetic Aperture Sonar (SAS) [1,2,3]. Each of these existing imaging methods has benefits, and disadvantages which cannot be solved constrained by the theory at the same time. Transducer designers must compromise considering their specific application requirements. Thus, many attempts are taken to improve the performance of different imaging methods [4,5,6,7]. Multibeam synthetic aperture sonar (MBSAS) is proposed and preliminarily researched as a novel imaging sonar technology, which combines the advantages of MBES and SAS to achieve a 3D full-scan detection with a high resolution [8,9]. In the along-track direction, MBSAS synthesizes a virtual aperture through the moving of the carrier that obtains a constant imaging resolution. In the across-track direction, beamforming is taken to estimate the direction and time of arrival (DOA and TOA) of the echo to obtain the image in the coordinate systems of angle and range (θ−r). Therefore, the imaging resolution and refinement mainly depend on the beamforming processing on the angle and range plane.

Based on our previous research, the MBSAS imaging can be divided into two independent steps as SAS processing in the along-track direction and beamforming in the across-track direction, which is a convenient benefit to the actual engineering applications. Conventional beamforming (CBF) is a classical and robust approach that is widely employed in the MBES system, and the analytic solution of the CBF employed by MBSAS imaging has also been researched. The receiving aperture limits the beamwidth of CBF that the resolution in the across-track direction also decreases enormously with the increasing range. The transducer designers must increase the amounts of elements to achieve a narrow beamwidth, but this choice performs uneconomical. Many spatial spectrum estimation approaches have been researched to achieve higher resolution with fewer elements, such as multiple signal classification (MUSIC), minimum variance distortionless response (MVDR), and many adaptive beamforming algorithms [10,11,12]. Nerveless, these beamforming algorithms achieve a higher resolution but at the sacrifice of the robustness in MBES systems, especially for the snapshot deficiency and low signal to noise ratio (SNR) applications. Yang proposed a novel deconvolved beamforming approach based on CBF and performs effectively in the low-frequency passive sonar system [13,14]. The deconvolved beamforming employs the point spread function (PSF) and CBF to recovery the theoretical azimuthal impulse, which gives consideration to the resolution and the robustness concurrently.

Deconvolved beamforming method may also perform as a reasonable attempt to increase the imaging performance of high-frequency sonar, especially for the 2D MBSAS with fewer elements in the across-track direction. However, the classical Richardson-Lucy (R-L) deconvolution method employed by Yang has a large amount of calculation with a low convergence velocity that limits the application of engineering [15,16]. In this paper, we propose a novel accelerated deconvolved beamforming method that introduces exponential acceleration and vector extrapolation to improve the convergence velocity of the classical R-L. Fast Fourier transform (FFT) is employed to increase the computation speed instead of the convolution and cross-correlation in the time domain. Theoretical analysis, simulation, tank and field experiments are taken on the 2D MBES transducer array structure we designed, to demonstrate the feasibility and advantages of the deconvolved imaging approach introduced into MBSAS. 3D high-accuracy level underwater acoustical surveying is achieved through this 2D transducer array system, which is also a great benefit to the 3D underwater model generation.

## 2. Echo Model and Imaging Theory of MBSAS

### 2.1. D Transducer Array and Echo Model of MBSAS

**Figure 1:**
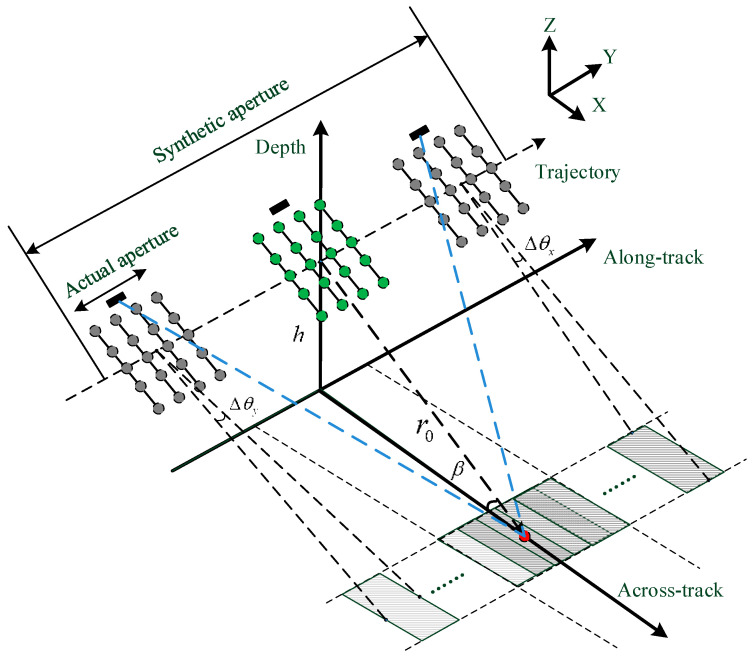
2D transducer array and echo model of MBSAS.

MBSAS combines the advantages of the MBES and SAS that the transducer array is specially designed to improve the imaging performance of the system. The rectangular array structure is employed in our design that we place four uniform linear arrays (ULA) in the along-track direction, with a distance larger than half wavelength to obtain a large receiving aperture. The transducer array can also be equivalently regarded as a 2D MBES, as shown in Figure 1. The multi-elements placement on the along-track can also improve the detecting efficiency that increases the limit of the carrier moving speed [17,18]. Although the large distance placement inevitably causes the grating lobe problem, we can just image in a narrow space in a single synthetic aperture processing period and splice the sub-images with the moving of the carrier. The width of the transmitting beam is designed larger than the traditional MBES system that the target is illuminated several times through a synthetic aperture processing period. The fundamental principle of SAS processing is that the coherence of echoes at different sampling positions must be guaranteed.

The 2D transducer array is employed for SAS processing in the along-track direction and MBES processing in the across-track direction. The resolution of the conventional 2D MBES system in the along-track direction is limited by the increasing footprint decided by beamwidth $\Delta \theta_{y}$ and range $r_{0}$. MBSAS employs the overlapped parts of the footprints that coherent integration is taken to the echoes at different sampling positions, as shown in Figure 1. A virtual transducer array is obtained through the moving of the carrier and has a constant along-track resolution. The transducer array performs as a ULA in the across-track direction after the SAS processing. Therefore, this research focuses on the beamforming in the across-track direction that finally determines the imaging resolution and the depth estimation accuracy of MBSAS.

### 2.2. Basic Imaging Algorithm of MBSAS

The imaging of MBSAS can be divide into two independent steps as SAS processing in the along-track direction and MBES processing in the across-track direction. SAS processing provides a complex sonar image which is obtained through the integral operation in a synthetic aperture processing period as Equation (1), where Γ is the synthetic aperture period, $f_{0}$ is the center frequency of the echo, s(t) is the received echo and τ is the time-delay at different sampling positions.

(1) $$I(r)=\frac{1}{\Gamma }\int_{-\frac{\Gamma }{2}}^{\frac{\Gamma }{2}}s(t)\exp(j2\pi f_{0}\tau )dt$$

Then, the SAS processing is extended to MBSAS and the time-delay can be expressed as Equation (2). The transmitter’s initial position is $\left(x_{0},y_{0},0\right)$ and the scanned target is located at $\left(x_{T},y_{T},z_{T}\right)$, the carrier moves at the speed of v, $\left(x_{k}(n),y_{k}(n),0\right)$ is the initial position of the nth element on the kth linear receiving transducer array. Therefore, the carrier’s moving time can be indicated as $\tau_{k}(n)$, which is also the echo time-delay, c is the acoustic speed.

(2) $$\begin{array}{ll} \tau_{k}(n) & =\frac{vy_{k}(n)-vy_{T}+c\sqrt{x_{0}^{2}-2x_{0}x_{T}+x_{T}^{2}+y_{0}^{2}-2y_{0}y_{T}+y_{T}^{2}+z_{T}^{2}}}{\left(c^{2}-v^{2}\right)}\\ & +\frac{\sqrt{-4\left(c^{2}-v^{2}\right)\left(\begin{array}{l} x_{0}^{2}-x_{k}^{2}(n)-2x_{0}x_{T}+2x_{k}(n)x_{T}\\ +y_{0}^{2}-y_{k}^{2}(n)-2y_{0}y_{T}+2y_{k}(n)y_{T} \end{array}\right)+{\left(-2vy_{k}(n)+2vy_{T}-2c\sqrt{\begin{array}{l} x_{0}^{2}-2x_{0}x_{T}+x_{T}^{2}\\ +y_{0}^{2}-2y_{0}y_{T}+y_{T}^{2}+z_{T}^{2} \end{array}}\right)}^{2}}}{2(c^{2}-v^{2})} \end{array}$$

The analytic form of SAS imaging has been derived by the previous research, with the bandwidth of LFM echo B as Equation (3).

(3) $$I_{SAS}(y,r)=\sin c(\frac{2f_{0}}{c}\frac{(y-y_{T})v\Gamma }{\sqrt{{\left(y-y_{T}\right)}^{2}+r^{2}}})\cdot \sin c(\frac{2B}{c}(r-r_{T}))\cdot \exp(j\frac{4\pi f_{0}}{c}(r-r_{T}))$$

After that, beamforming is taken in the across-track direction based on the ULA to indicate the DOA and TOA of the echoes, and we have derived the analytical solution of CBF imaging as Equations (4) and (5).

(4) $$\begin{array}{ll} I_{MBSAS}(y,r,\theta ) & =\sum_{n=0}^{N-1}I_{SAS}(y,r+c\tau (n),n)\\ & \approx \sin c(\frac{2f_{0}}{c}\frac{(y-y_{T})v\Gamma }{\sqrt{{\left(y-y_{T}\right)}^{2}+r^{2}}})\cdot \sin c(\frac{2B}{c}(r-r_{T}))\exp(j(\frac{4\pi }{\lambda }(r-r_{T}))\cdot I_{MBES}(\theta ) \end{array}$$

(5) $$I_{MBES}(\theta )=\frac{\sin(\frac{2N\pi d}{\lambda }(\sin\theta -\sin\theta_{T}))}{\sin(\frac{2\pi d}{\lambda }(\sin\theta -\sin\theta_{T}))}\;\cdot \exp(\frac{\left(N-1)\pi d(\sin\theta -\sin\theta_{T}\right)}{\lambda })$$

where τ(n)=sinθ⋅nd/c is the nth time-delay of the element. The average element spacing d and the number of elements N indicates the manifold of the transducer array, λ is the wavelength of the echo, and d=λ/2 as designed. θ is the preset beam angle and $\theta_{T}$ is the beam angle of the target. In this paper, research is taken based on the 2D transducer array we designed that has the rectangular structure of 4×32.

Equations (4) and (5) indicate that the 3D imaging in the 3D coordinate system of y−r−θ can be divide into two independent steps as SAS processing (y−r) and MBES processing (θ−r), which are both 2D processing. The first item of Equation (5) indicates the amplitude of the image, and the second item of Equation (5) indicates the phase shift between adjacent elements. The amplitude performs similarly to the PSF of a ULA but with twice the variation. The reason is that the SAS processing transmits and receives echoes at each sampling position to synthesize signal coherently, which is different from the single transmission with multiple receiving of moving 2D MBES.

## 3. Deconvolved Beamforming and Accelerated R–L Algorithm

### 3.1. Directivity and CBF of a ULA

In the applications of MBES, CBF is widely employed as a robust approach with little computation. The main-lobe width and sidelobe level are limited by the array manifold, which is equivalent to the PSF of the specific transducer array. Given a ULA which has N elements with a spacing of d, when the target locates at the direction of ϑ, the directivity function can be expressed as Equation (6). θ is the angle between the target and the normal, which is also defined as the beam angle in MBES.

(6) $$R(\theta |\vartheta )={\left|\frac{\sin\left(\frac{N\pi d}{\lambda }(\sin\theta -\sin\vartheta )\right)}{N\sin\left(\frac{\pi d}{\lambda }(\sin\theta -\sin\vartheta )\right)}\right|}^{2}$$

The CBF output of a ULA can be coherently accumulated from the received signal s(t) and the weighted vector w(θ) as

(7) $$P_{CBF}(\theta )=\frac{1}{N^{2}}w^{H}(\theta )s(t)s^{H}(t)w(\theta )$$

(8) $$w(\theta )=[1\;e^{-j\frac{2\pi }{\lambda }\cdot 1d\sin\theta }\;e^{-j\frac{2\pi }{\lambda }\cdot 2d\sin\theta }\ldots e^{-j\frac{2\pi }{\lambda }\cdot (N-1)d\sin\theta }]$$

The beamforming processing can be regarded as the integration of the product of PSF and the angle impulse, which is equivalent to the convolution process of the linear system. The limiting condition of the convolution is that the impulse response of the system should be linear shift-invariant, which cannot be achieved directly from a ULA [19]. The linear shift-invariant can be obtained as R(sinθ|sinϑ)=R(sinθ−sinϑ) through a transformation that the convolution is expressed as Equation (9).

(9) P(sinθ)=∫R(sinθ|sinϑ)S(sinϑ)dsinϑ=R(sinθ)∗S(sinθ)

### 3.2. Deconvolved Beamforming and Accelerated R–L Algorithm

Deconvolved beamforming is the reverse operation that employs the CBF output and the PSF of the transducer array to recovery the ideal echo source impulse through the iterative process. Several deconvolved beamforming approaches have been researched in the fields of optics, radar imaging, and radio astronomy, such as DAMAS, NNLS, FISTA, and R-L. DAMAS can be employed either in the linear or nonlinear shift-invariant system. However, the efficiency and computational precision of DAMAS barely satisfactory the sonar system, which usually has dozens or even hundreds of elements such as MBES [20]. The main computation of DAMAS focuses on the iterative solution of linear equations through the Gauss-Seidel method [21]. The limiting condition of this method is the matrix should be diagonally dominant or symmetric positive definite matrix, which is hardly guaranteed in the underwater acoustical imaging [22]. NNLS and FISTA are iterative methods based on the least square principle that have been employed in the field of noise source’s location [23,24,25]. R-L is an iterative method based on the maximum likelihood estimation theory with non-negativity constraints which has a fair recovery and high-resolution effect. In Yang’s research, R-L is proved to improve the resolution of CBF and applied in the low frequency passive sonar and circular array.

In a linear shift-invariant system, the beamforming processing can be expressed by a convolution as Equation (10) that h is the impulse response of the system (PSF of the transducer array), x is the input of the system (target direction to be solved), n is the system noise, and y is the output of the system (CBF output). ⊗ is defined as the convolution process.

(10) y=h⊗x+n

In Yang’s research [13], classical R-L process can be achieved through the iteration that described by the integral as Equation (11)

(11) $$\begin{array}{ll} s^{k+1}(\sin\theta ) & =s^{k}(\sin\theta )\int_{-\infty }^{\infty }\frac{h(\sin\vartheta -\sin\theta )\cdot r(\sin\vartheta )}{\int_{-\infty }^{\infty }h(\sin\vartheta -\sin\theta )\cdot s^{k}(\sin\theta )d\sin\theta }d\sin\vartheta \\ & =s^{k}(\sin\theta )\int_{-\infty }^{\infty }h(\sin\vartheta -\sin\theta )\cdot \frac{r(\sin\vartheta )}{r^{k}\sin(\vartheta )}d\sin\vartheta \end{array}$$

where $r^{k}(\sin\vartheta )=\int_{-\infty }^{\infty }h(\sin\vartheta |\sin\theta )s^{k}(\sin\theta )d\sin\theta$, k denotes the iteration number.

The iterative sequence $s^{k}(\sin\theta )$ can be shown to converge to the solution that satisfies Equation (12)

(12) $$\underset{k\to \infty }{\lim}s^{k}(\sin\theta )=\underset{s(\sin\theta )}{\mathrm{argmin}}L(r(\sin\vartheta ),r^{k}(\sin\vartheta ))$$

where $L(r(\sin\vartheta ),r^{k}(\sin\vartheta ))$ is the Csiszar discrimination given by

(13) $$L(r(\sin\vartheta ),r^{k}(\sin\vartheta ))=\int_{-\infty }^{\infty }r(\sin\vartheta )\log\frac{r(\sin\vartheta )}{r^{k}(\sin\vartheta )}d\sin\vartheta -\int_{-\infty }^{\infty }\left|r(\sin\vartheta )-r^{k}(\sin\vartheta )\right|d\sin\vartheta$$

Classical R-L process can also be simply described for the engineering application as the iterative process of Equation (14), where ${\hat{x}}_{k}$ is the estimated value at the kth iterative process,⋅ is defined as the scalar multiplication, ∗ is defined as the correlation operation.

(14) $${\hat{x}}_{k+1}={\hat{x}}_{k}\cdot h*\left[\frac{y}{h⊗{\hat{x}}_{k}}\right]$$

There are two problems with the R-L algorithms that must be solved when we apply R-L to engineering applications. The first is the computational complexity, which limits the real-time performance of the imaging algorithm. The second is the convergence velocity that we are eager to achieve a narrower beam with fewer iterations. It is shown in Equation (14) that the main computational complexity focuses on the convolution and correlation operations where the process can be converted to the frequency domain and employs the FFT method. The limiting condition of FFT is the linear shift-invariant system, and it is also the preconditions of the R-L algorithm. With the development of processers, FFT can be implemented efficiently on the FPGA (Field Programmable Gate Array) together with CBF, as we have achieved in our produced MBES [26].

Several acceleration algorithms are proposed to improve the convergence performance of the classical R-L. Although the computational complexity of a single iteration is increased, the convergence velocity has also been improved that the accelerated algorithms are more suitable to be employed in the engineering applications [27,28]. Exponential correction parameter is introduced into classical R-L as an acceleration factor that has a better convergence performance as Equation (15). The exponential correction parameter should be set as 1≤p≤3 to balance the convergence velocity and stability of imaging.

(15) $${\hat{x}}_{k+1}={\hat{x}}_{k}\cdot h*{\left[\frac{y}{h⊗{\hat{x}}_{k}}\right]}^{p}$$

A method based on the vector extrapolation is proposed to improve the convergence velocity which calculates the increment between the current and the previous iterations, as shown in Equations (16) and (17).

(16) $${\hat{x}}_{k+1}=\Delta_{k}\cdot \left(h*\left[\frac{y}{h⊗\Delta_{k}}\right]\right)$$

(17) $$\{\begin{array}{l} \alpha_{k}=\left[\frac{\sum \left({\hat{x}}_{k}-\Delta_{k-1})\cdot ({\hat{x}}_{k-1}-\Delta_{k-2}\right)}{\sum \left({\hat{x}}_{k-1}-\Delta_{k-2})\cdot ({\hat{x}}_{k-1}-\Delta_{k-2}\right)}\right]\\ \Delta_{k}={\hat{x}}_{k}+\left|\alpha_{k}\right|\cdot ({\hat{x}}_{k}-{\hat{x}}_{k-1}) \end{array}$$

Compared with the algorithms above, the classical and exponential acceleration methods employ the current iteration that the convergence performance is limited. The vector extrapolation method employs the current and previous iterations to calculate the acceleration factor through the direction gradient that has a better convergence velocity. In this paper, we combine the exponential acceleration and vector extrapolation method that achieve a narrower beam with fewer iterations, and the processing time increased finitely. The exponential parameter is introduced to modify the iterative process, which accelerates the convergence process as Equation (18). Not only the iterative function but also the vector extrapolation is controlled by exponential parameter to manage the convergence velocity. FFT is employed as the acceleration method of convolution and correlation operations. The convergence velocity r, performed in the vector extrapolation α, is controlled as 0.5≤r≤1, the exponential correction parameter should be set as 1≤p≤3.

(18) $${\hat{x}}_{k+1}=\left[{\hat{x}}_{k}+\left|\alpha_{k}\right|\cdot ({\hat{x}}_{k}-{\hat{x}}_{k-1})\right]\cdot {\left\{\mathrm{ifft}\{\mathrm{fft}(\bar{h})\cdot \mathrm{fft}(\left[\frac{y}{\mathrm{ifft}\{\mathrm{fft}(h)\cdot \mathrm{fft}(\left[{\hat{x}}_{k}+\left|\alpha_{k}\right|\cdot ({\hat{x}}_{k}-{\hat{x}}_{k-1})\right])\}}\right])\}\right\}}^{p}$$

(19) $$\alpha_{k}={\left[\frac{\sum \left({\hat{x}}_{k}-\Delta_{k-1})\cdot ({\hat{x}}_{k-1}-\Delta_{k-2}\right)}{\sum \left({\hat{x}}_{k-1}-\Delta_{k-2})\cdot ({\hat{x}}_{k-1}-\Delta_{k-2}\right)}\right]}^{r}$$

**Figure 2:**
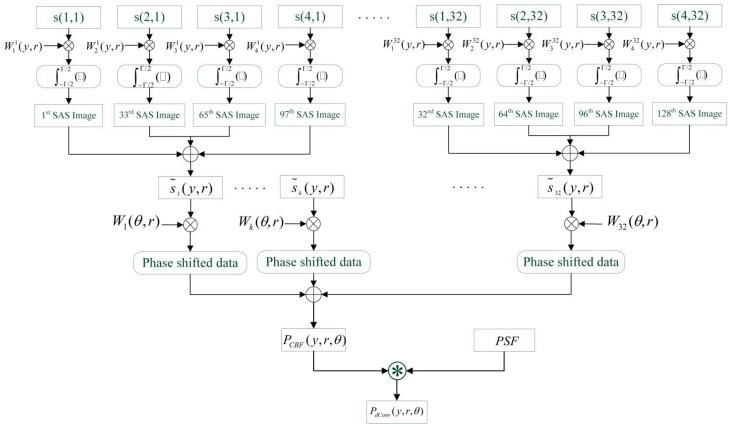
Processing scheme of the 3D MBSAS imaging system.

The processing scheme of the 3D MBSAS imaging system is shown in Figure 2, which is divided into two parts as the SAS processing in the along-track direction and the beamforming in the across-track direction.

An integral operation is taken in the whole synthetic aperture period, and each element achieves a 2D complex SAS image. The weight $W_{k}^{n}(y,r)$ is calculated through the time delay between the transmitter and the receivers, considering the position of the elements, the moving speed, and the scanned points. A weighted summation is taken by each subarray, and a virtual element is obtained. Therefore, a virtual ULA similar to an MBES is generated so that the beamforming process can be conducted. Phase shift beamforming then takes place, and the weight is calculated through the preset beam angle and the range. A weighted summation of the phase-shifted data is performed on the 32 elements, and a 3D MBSAS image is obtained as the output of CBF. After that, PSF is calculated based on the directivity function of the 32 elements virtual ULA, which is equivalent to the CBF output when the target locates at $\theta =0^{\circ }$. Accelerated R–L algorithm is carried based on the CBF and PSF that the process should be taken at each sampling interval to keep the real-time of imaging, as we have achieved in MBES.

## 4. Imaging Algorithms Simulations

**Figure 3:**
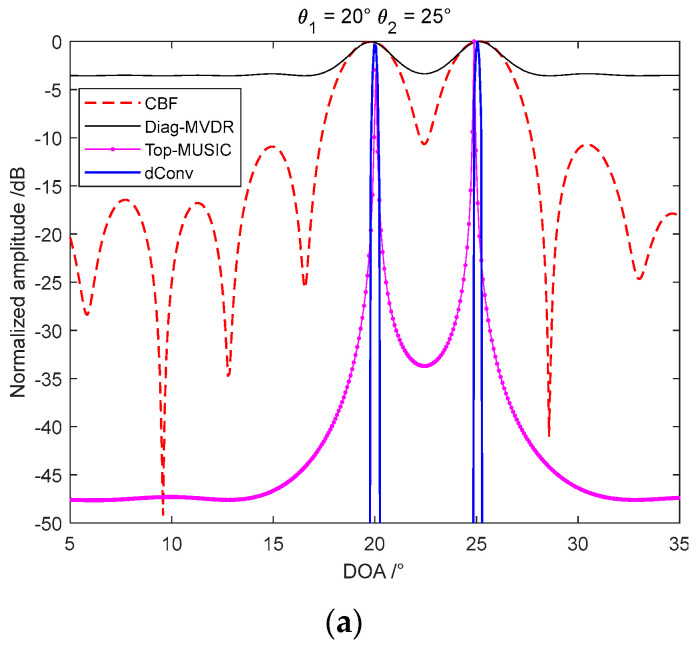

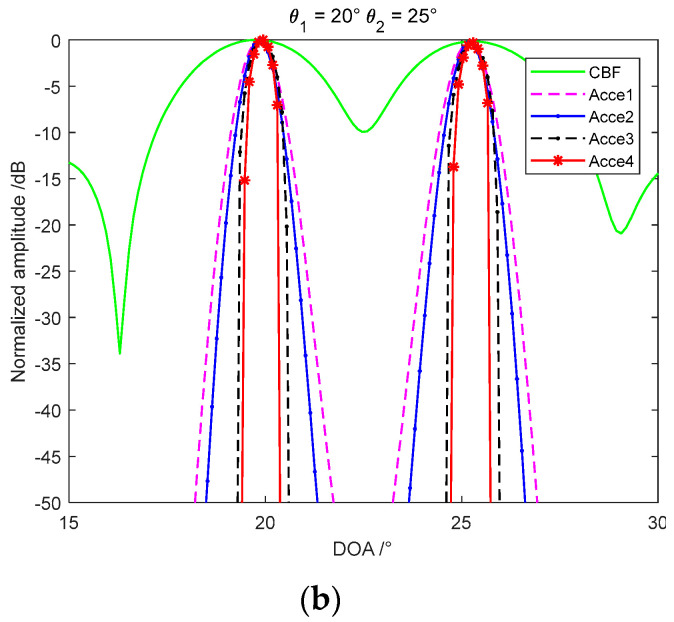
Imaging performance of deconvolution beamforming: (**a**) imaging comparison between different algorithms; and (**b**) imaging performance of different acceleration methods.

The advantage of the deconvolution beamforming algorithm is that it can achieve a narrower beam with a robust performance as CBF, especially for the coherent imaging targets of MBSAS. MUSIC and MVDR are taken as the comparative methods first to demonstrate the performance of deconvolution beamforming. We locate two coherent targets at $\theta_{1}=20^{\circ }$ and $\theta_{2}=25^{\circ }$, there are 32 elements locate in the direction of across-track, and the center frequency of the signal $f_{0}=150\;\mathrm{kHz}$. Single snapshot is taken as the environment of active imaging sonar performs time-variant, which is different from the passive location system. As classical MUSIC and MVDR performs barely satisfactorily when imaging some coherent targets, that the Toeplitz-MUISC and diagonal loading MVDR are taken as the comparison algorithms [29,30,31]. It is shown in Figure 3a that the CBF has a wide beamwidth and high sidelobe level, limited by the actual receiving array aperture. Toeplitz-MUISC performs a high-resolution imaging method with narrow beamwidth and low sidelobe level that break through the Rayleigh-limit. Although the diagonal loading MVDR can separate the dual coherent targets, the beamwidth and sidelobe level performance have lost the advantages of high-resolution imaging methods. The main computation of MUSIC and MVDR is the singular value decomposition (SVD) and the matrix inversion that are time-consuming processes. The deconvolution beamforming algorithm has an almost equivalent beamwidth and better sidelobe level than MUSIC. Furthermore, the deconvolution beamforming is taken based on the output of CBF and PSF which evades the source amounts and coherent interference problems, which also has a better robust performance than other high-resolution spatial spectrum estimation methods.

The convergence performance of different acceleration methods is shown in Figure 3b that they take iterations with an FFT process to improve the computation speed. Acceleration algorithms 1 and 2 both take 60 times iterations; 3 and 4 both take 20 times iterations. Acceleration algorithm 1 is taken as the classical R-L of Equation (14), which has already performed an appreciable improvement than CBF with a narrower beam and lower sidelobe. Acceleration algorithm 2 is taken by the exponential correction parameter p=1.5 in Equation (15) that has a better convergence velocity. Acceleration algorithm 3 is the imaging result of the vector extrapolation method that has a significant acceleration improvement than the exponential correction method. Acceleration algorithm 4 is the method we proposed in this paper that the vector extrapolation and the iterative function are both accelerated by the exponential parameter. The acceleration algorithm 4 has a narrower beamwidth than acceleration algorithm 3, which means the better convergence velocity. The only disadvantage of this method proposed is the exponentiation increases the computation that we compared the processing time of different acceleration methods. Furthermore, as the convergence velocity decreases seriously with the increasing iterative number, we can choose the finite iterations which satisfy the requirement in actual engineering applications. Although acceleration with algorithm 3 and 4 take 1/3 times the iterations of algorithm 1 and 2, they still achieve a better performance than the classical methods.

**Figure 4:**
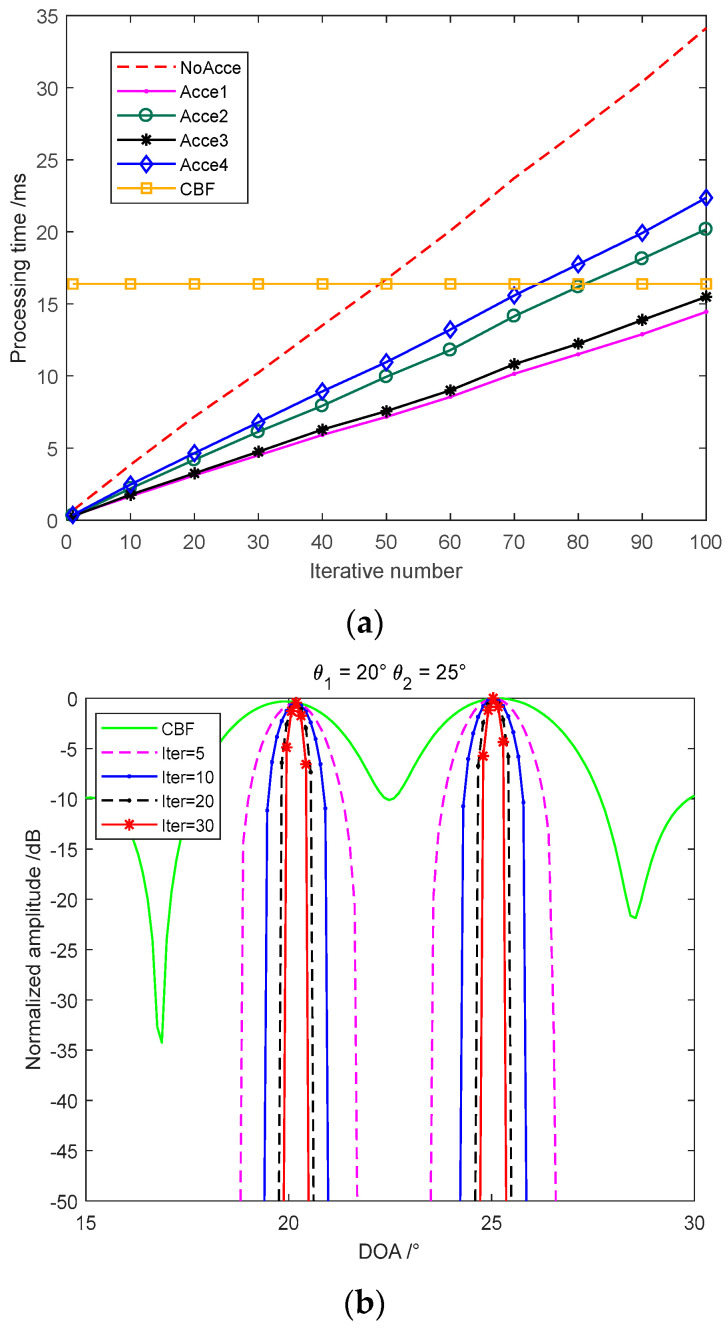
Processing time of different algorithms and the beamwidth with the increasing iterative number: (**a**) processing time of different algorithms; and (**b**) imaging beamwidth with the increasing iterative number.

The processing time of different acceleration algorithms is shown in Figure 4a. We take 200 times Monte-Carlo experiment to obtain 1024 beams and employ the average value by MATLAB, Core i7 @ 2.8 GHz (single processing core used). The classical R-L based on the time-domain convolution and correlation operation takes the most processing time with no acceleration effect. Acceleration algorithm 1 takes FFT instead of time-domain processing that has the least processing time. Acceleration algorithm 2 takes the exponential correction parameter as p=1.5 which increases the calculation. Acceleration algorithm 3 employs the current and previous iterative results to estimate the extrapolative vector that increases the processing computation.

Although the acceleration algorithm 4 takes the most time in single processing, the advantage of fast convergence decreases the iterative number so that the total processing time is still acceptable in the actual applications. The main computation is the FFT that can be implemented efficiently on the FPGA together with CBF, as what we have achieved in our produced MBES in the sampling interval in real-time. The processing time of the deconvolution beamforming is less than the single CBF processing when we just choose dozens of iterations. The total processing time performs acceptably in the actual engineering applications. Figure 4b shows that the convergence performance slowed down with the increasing iterative number. The iterative number simulation indicates that the acceleration algorithm we proposed can achieve a narrow beamwidth in 10–20 times iteration that has already satisfied the engineering application and kept the real-time imaging.

**Figure 5:**
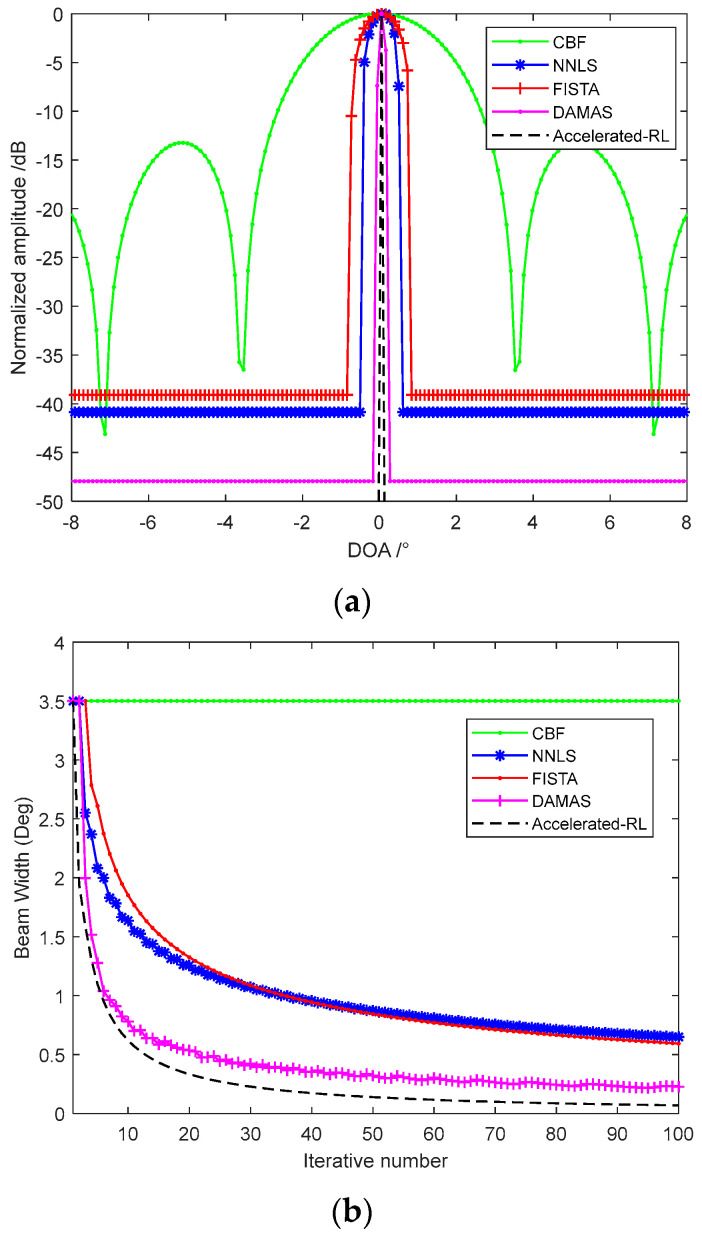
Imaging performance of different deconvolution beamforming methods: (**a**) imaging performance of different methods; and (**b**) imaging beamwidth with the increasing iterative number.

We also compare the imaging performance of different deconvolution beamforming methods. In Figure 5a, 100 times iteration is taken to compare the different methods. The results indicate that NNLS and FISTA have a wider beam width than others. Although the FISTA introduces the shrinkage-thresholding theory to increase the gradient descent speed and reduces the processing steps, the least-squares principle limits the convergence rate. DAMAS has a better beam width at the same iteration times, which employs the Gauss-Seidel method to take the deconvolution processing. Accelerated R-L is an iterative method based on maximum likelihood estimation theory that has the best performance. The Accelerated R-L iteration can be terminated by a preset residual threshold.

The beamwidth at different iteration times is shown as Figure 5b that the convergence rate all slows down obviously with the iteration times increasing. NNLS and FISTA have the lowest convergence rate and trends to be stable with the iteration times increasing. DAMAS has a better convergence rate, and the beamwidth decreases obviously with the first dozen iterations. Accelerated R-L has the best convergence rate, so that the beam width is decreased to less than 0.5° in 20 iterations, which is acceptable for engineering applications.

**Figure 6:**
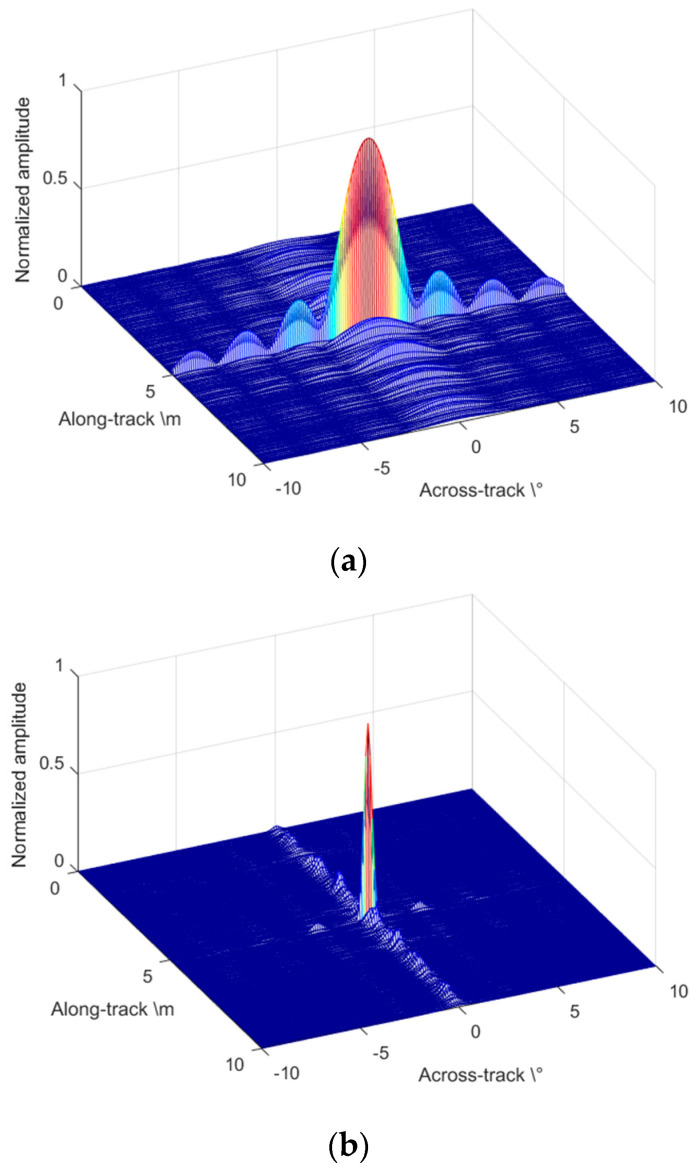
Imaging comparison of the single point target in the across-track direction: (**a**) slice of CBF; and (**b**) slice of accelerated deconvolved beamforming.

Single point imaging simulation is taken as shown in Figure 6 to compare the performance of the CBF and the proposed accelerated deconvolved method. SAS processing is taken first to obtain the same complex images, which have a constant resolution in the along-track direction. In Figure 6a, CBF is taken in the across-track direction that the imaging main lobe beamwidth reaches $\Delta \theta =3.5^{\circ }$, limited by the actual receiving aperture. The sidelobe of CBF also performs with a high level and the energy leaks to the along-track direction that causes the whole image blurring. Accelerated deconvolved beamforming achieves a narrow beamwidth with a low sidelobe level in 10 iterations. The images are sliced shown on the same depth plane as Figure 6b, which indicates the beamwidth and sidelobe in the along-track and across-track directions. The imaging comparison indicates that the accelerated method performs an effective method to increase the imaging resolution of MBSAS in the across-track direction. Furthermore, the proposed accelerated deconvolved beamforming has a better energy focusing ability than that of CBF, which is an excellent benefit to the underwater acoustic 3D high-resolution imaging system.

Solid targets are also simulated in this section to demonstrate the imaging performance of the MBSAS deconvolved beamforming algorithm, including the cubical target and the complex geometry target. The target echo is generated based on the model of acoustical highlights, which separates the complex targets into hundreds of highlights on the surface. For example, a cubical target is divided by the surface tangent and each element receives the echo superposed by all the highlights. The analytical solution of the position and the time delay of the cubical target highlights can be solved that the echoes are generated. Solid target simulation parameters are shown in Table 1 that we locate a cubical target at the flat seabed of 16 m.

**Figure 7:**
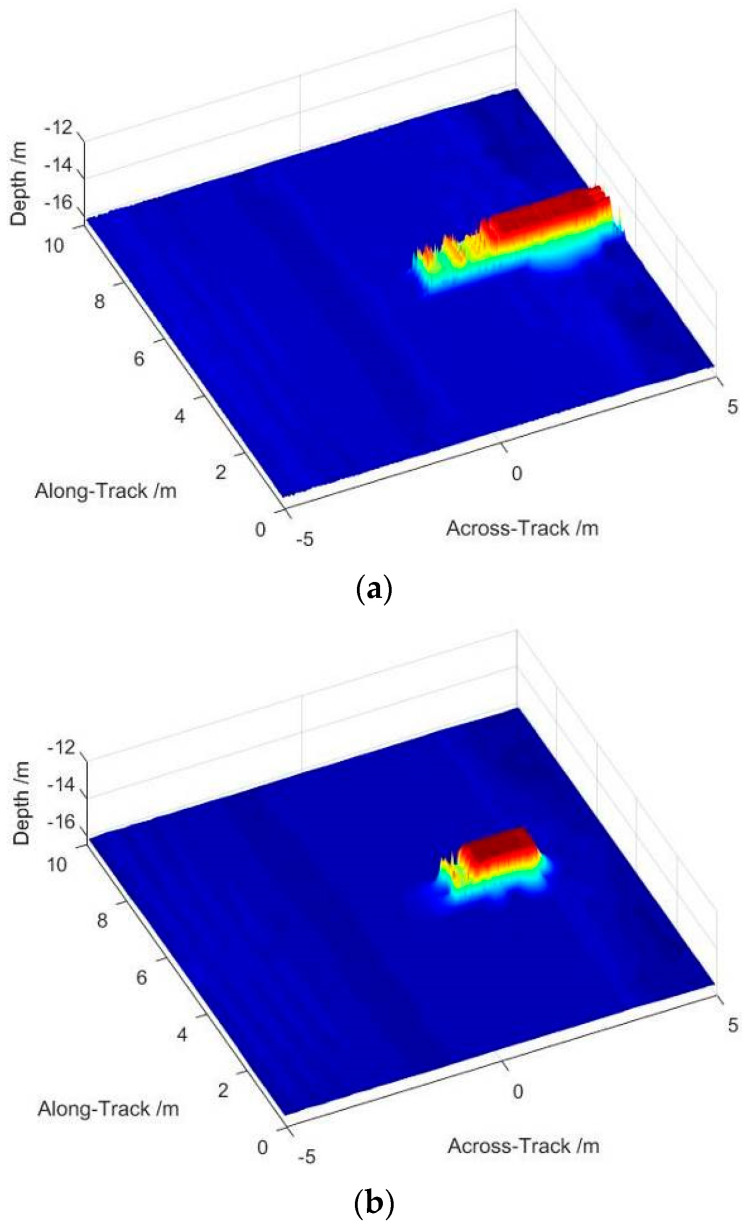
Imaging result of cubical target by different methods: (**a**) imaging result of CBF in the across-track direction; and (**b**) imaging result of accelerated deconvolved beamforming in the across-track direction.

Imaging comparison between the CBF and accelerated deconvolved beamforming is shown in Figure 7. TOA and DOA of the 3D image are estimated and gridding displayed, which is equivalent to the thinning that makes the images have better visibility. The resolution in the along-track direction keeps constant as the beamforming and the SAS processing can be separated into two individual steps. The imaging size of the cubical target expands inevitably by CBF in the across-track direction as it is limited by the actual receiving aperture, shown in Figure 7a. Figure 7b displays the imaging output of the accelerated deconvolved beamforming that indicates the accurate target location. The size of the target in the across-track direction is detected approximately to the actual situation, owing to the narrow beamwidth and the low sidelobe level.

**Figure 8:**
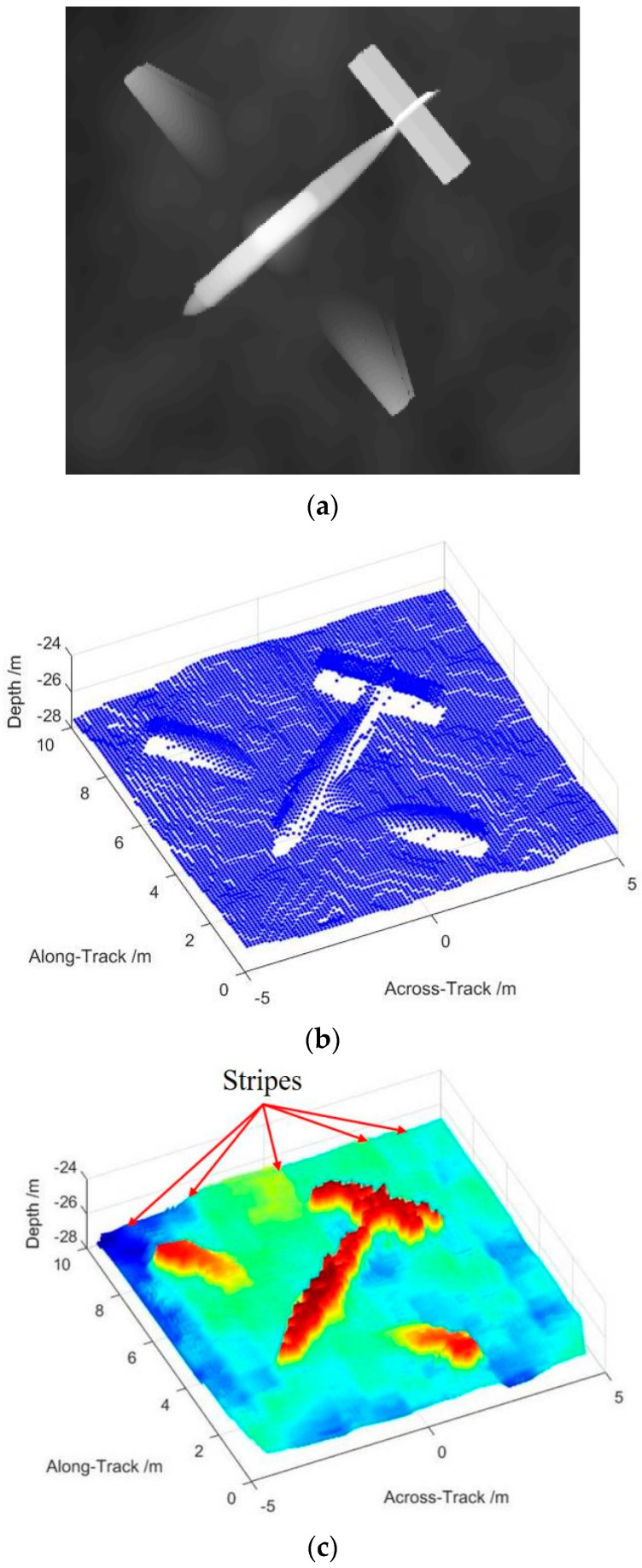

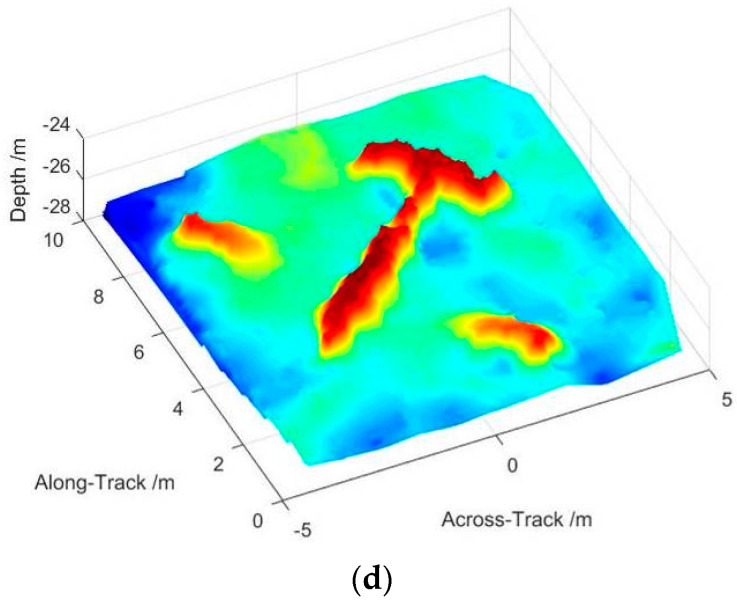
Complex geometry target simulation: (**a**) original grayscale image of airplane; (**b**) highlights generated from SFS method; (**c**) imaging result of CBF with stripes; and (**d**) imaging result of accelerated deconvolved beamforming.

To the complex geometry target, it’s hard to describe the highlights on the surface as an analytical solution that we employ the SFS (Shape from Shading) algorithm to generate the echoes as an accessible method [32,33]. We also simulated a complex geometry target as an airplane from a grayscale image by the SFS method. The original grayscale image has the size of 320×320 pixels as Figure 8a and we extend it to the highlights with the size of 10 m×10 m at a depth of 28 m as Figure 8b.

SAS processing in the along-track direction is taken first and then the beamforming in the across-track direction is taken to obtain the 3D image. The imaging result of CBF is shown in Figure 8c that we can observe the CBF processing achieves the basic 3D imaging. The image indicates the correct position and size of the preset target, where the airplane and the seabed are separated obviously. Nevertheless, the details of the image perform not as perfect as considered. The main body of the airplane manifests as rough, and the seabed has a visible striped image. This situation is caused by the wide beamwidth and the sidelobe level that generate a wide beam footprint. The accelerated deconvolved beamforming has effectively improved these situations that achieve a more detailed 3D image as shown in Figure 8d. The main body of the airplane performs smoothly and there is no conspicuous stripe on the seabed. The imaging result of accelerated deconvolved beamforming matches the highlights of Figure 8b well, as this method supplies a narrow beamwidth and low sidelobe level which is a great benefit to display the details. The stripe on the seabed is also removed as the footprints are tinny and discontinuous, owing to the narrow beamwidth.

## 5. Experiment and Results

### 5.1. Field Experiment of Deconvolved Beamforming Applied on MBES

MBES is the research foundation of MBSAS 3D high-resolution imaging that we employ the deconvolved beamforming on the MBES first to demonstrate the imaging performance. We carried the field experiment in the sea around Qingdao for the extensive area surveying, based on the MBES system designed and produced by our lab. We choose the seabed area of 60 m and survey in a broad swath width to evaluate the improvement of the method proposed with the parameters in Table 2.

**Figure 9:**
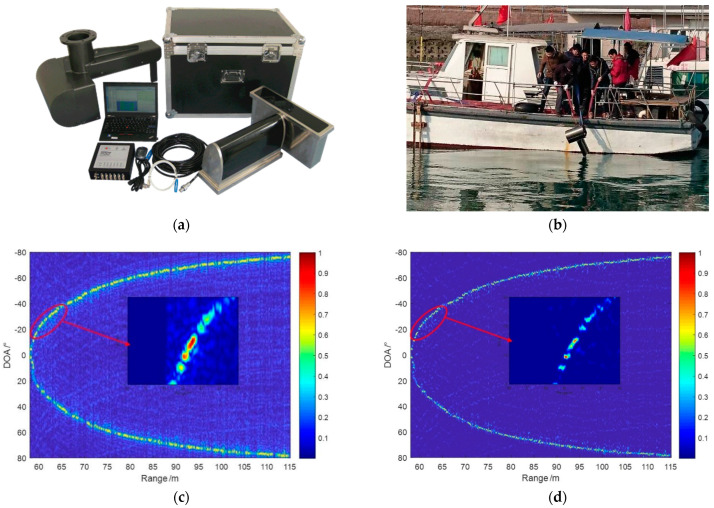
Field experiment carried in Qingdao area with the MBES: (**a**) the horseshoe-shaped MBES equipment; (**b**) MBES installation of the field experiment; (**c**) seabed survey result of CBF; and (**d**) seabed survey result of accelerated deconvolved beamforming.

Figure 9a shows the MBES we designed, which has a horseshoe-shaped transmitting transducer array. This unique structure can enhance the echo energy at the large beam angles, which has the directivity likes a horseshoe. The MBES employs a ULA as the receiving array which has the directivity as Equation (6). We install the MBES equipment at the sideboard far away from the engine to avoid the propeller noise and bubble interference, as shown in Figure 9b. The transducer is fixed 50 cm below the surface to avoid the wave bubble and vibration of mounting rod interference. Beamforming is taken from −80° to 80°, which realizes an ultra-wide area coverage. 2D image is obtained through the beamforming that indicates the DOA and range, which is also called the water column images of MBES. Typically, we estimate the TOA from the preset beam angle images and generate the depth information. Here, we focus more on the imaging comparisons that the beamforming is normalized to enhance the images at each sampling interval and local amplificated to show more details.

Figure 9c indicates the imaging result of the seabed through CBF. The seabed image is evident from the background and has high energy centrality. However, the beamwidth is limited that the discrimination of adjacent targets performs poorly. The background of the image is disturbed by the noise, which is caused by the high sidelobe level. Furthermore, the tunnel effect appears owing to the main lobe energy leaking, as shown in the local amplificated image. The accelerated deconvolved beamforming proposed improves these situations effectively, as shown in Figure 9d. A narrow beamwidth is achieved through 10 times iterations that the adjacent targets can be separated obviously. The energy centrality is also improved that the highlights focus on the main lobe. The tunnel effect is eliminated, and the background noise is reduced owing to the narrow beamwidth and the low sidelobe level.

**Figure 10:**
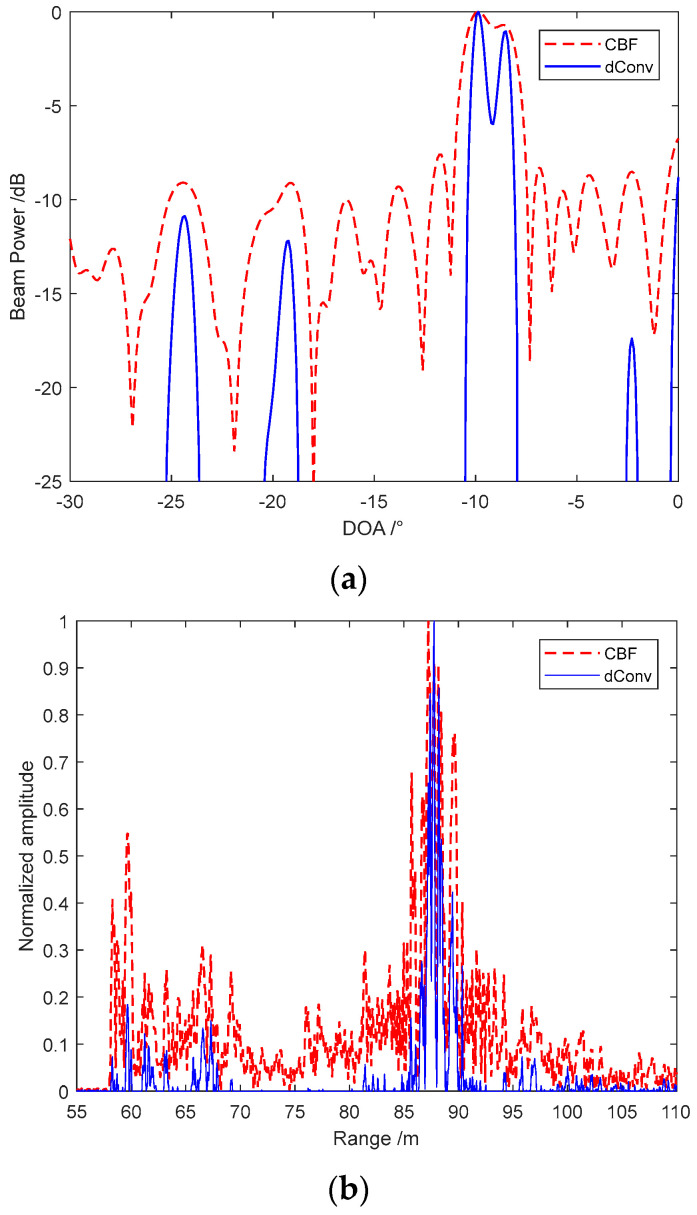
Sliced comparison between CBF and accelerated deconvolved beamforming: (**a**) beamwidth comparison between CBF and accelerated deconvolved beamforming; and (**b**) echo energy comparison between CBF and accelerated deconvolved beamforming.

Furthermore, we sliced the image to compare the beamwidth and echo energy ability of different methods. The accelerated deconvolved beamforming achieves a narrow beamwidth that the adjacent targets can be separated obviously, compared with the aliasing image at the selected sampling moment shown in Figure 10a. As the CBF has a wide beamwidth and high-level sidelobe that we cannot distinguish the sidelobe and the weak peak. The accelerated deconvolved beamforming has a low level sidelobe to distinguish several weak peaks from the sidelobes that achieves obvious improvement than CBF. We also select the beam amplitude at $\theta =45^{\circ }$ to compare the echo energy from different methods as Figure 10b. Weak false peaks appear at 60 m of CBF that is disturbed by the tunnel effect, which may lead to the depth estimation error of MBES.

Furthermore, the main lobe echo extends and accepts more directional echoes, which is limited by the beamwidth and sidelobe level. The accelerated deconvolved beamforming method has a better tunnel effect rejection capability that the false peaks are decreased effectively. Moreover, the method proposed has a better energy focusing ability that the echo from other directions is suppressed and the echo expansion is also decreased. The field experiment comparisons indicate that the accelerated deconvolved beamforming proposed can achieve a narrow beam with low sidelobe and keeps the robustness as CBF, which is an excellent benefit to imaging sonar systems. The possibility of the method’s application to MBSAS is proved preliminarily through the experiment.

### 5.2. Tank Experiment of Deconvolved Beamforming Applied on MBSAS

Based on the research foundation of MBES, we take the tank experiment to demonstrate the performance of deconvolved beamforming applied on the MBSAS system, which is designed and produced by our lab. The transducer array is specially designed with a rectangular structure that is equivalent to a 2D MBES, which has an along-track element spacing of 20 times the half-wavelength. We employ two balls as the small target to evaluate the imaging performance of MBSAS. LFM signal is introduced to achieve a high time-delay resolution, together with the pulse compression employed as the pre-processing, tank experiment parameters are shown as Table 3.

**Figure 11:**
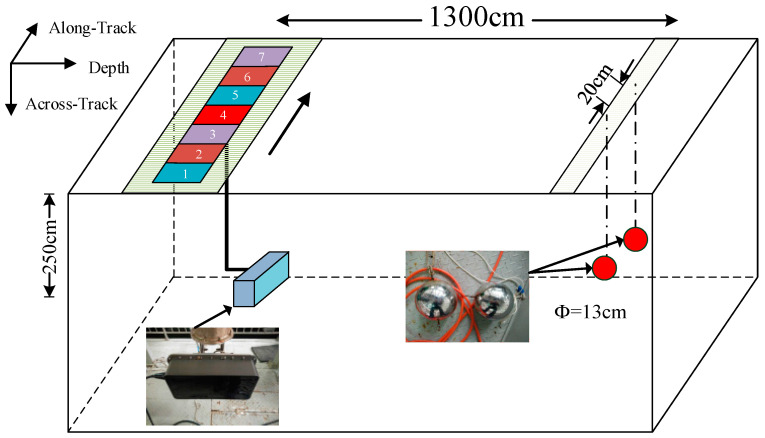
Dual balls target imaging experiment of MBSAS.

The diameter of each ball is 13 cm, and the distance between the centers is 20 cm, which cannot be separated by CBF in theory. We locate the targets in a long-distance as 13 m to generate a large footprint to compare the imaging performance. The experimental site and the trajectory of the transducer array are shown in Figure 11. The transducer array is rotated 90° to achieve a long-distance detecting, considering the experiment tank situations. Seven sampling positions are selected to synthesis the virtual aperture with an interval of 15 cm, twice the transmitter aperture. Therefore, the virtual aperture of this tank experiment reaches 105 cm, which is 2.5 times of the actual aperture of the receiving array.

**Figure 12:**
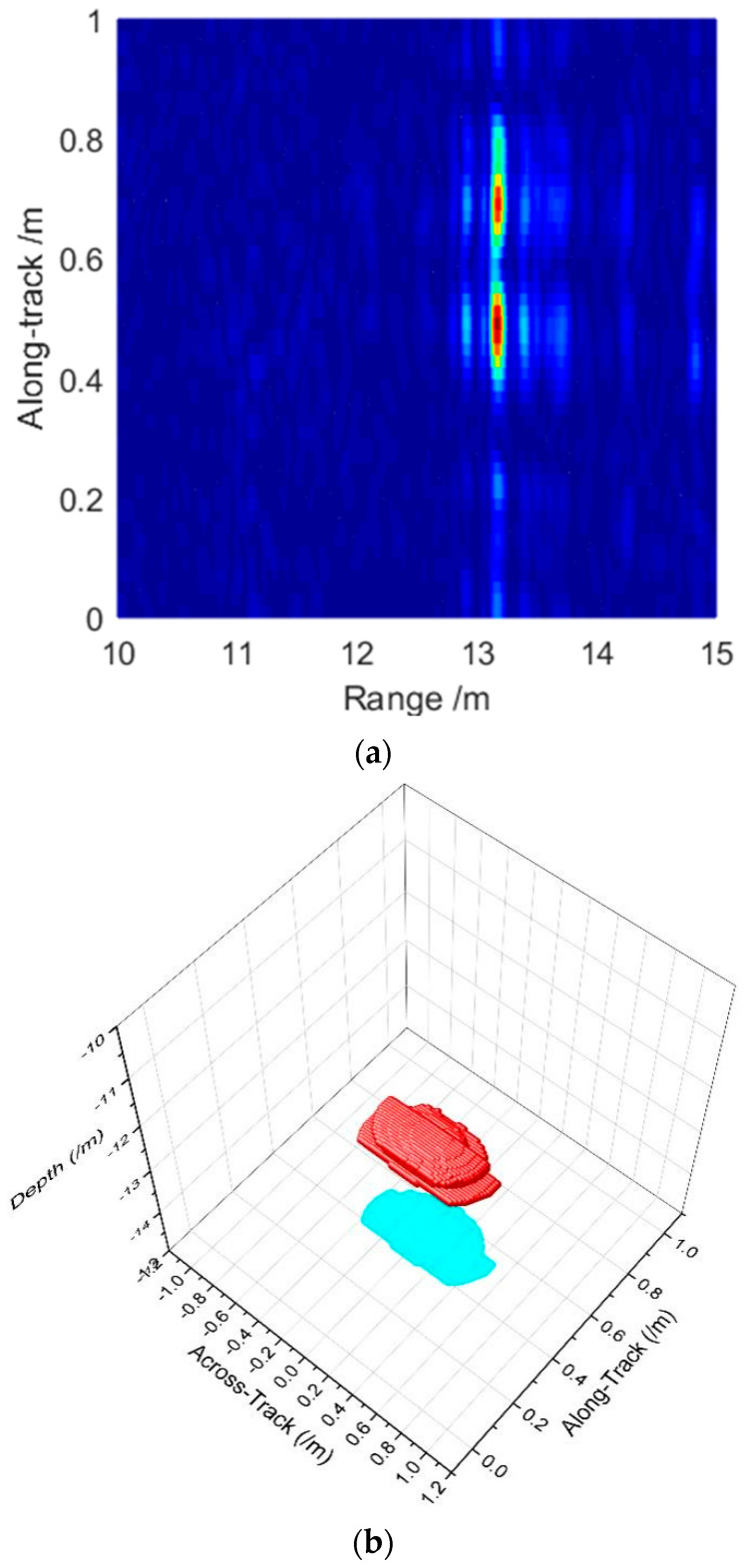

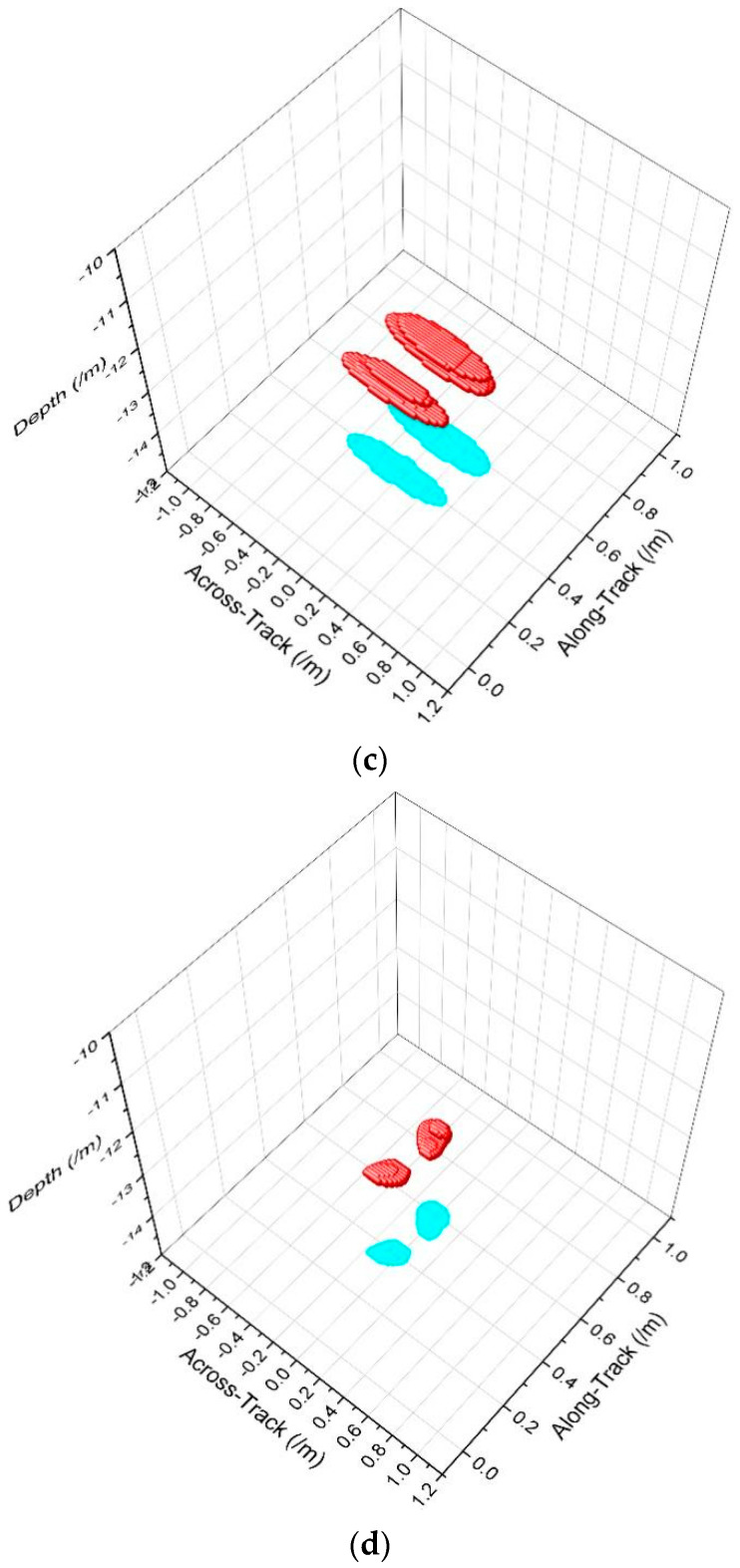
Imaging comparison between different methods: (**a**) 2D imaging result of SAS; (**b**) imaging result of MBES; (**c**) imaging result of MBSAS with CBF in the across-track direction; and (**d**) imaging result of MBSAS with accelerated deconvolved beamforming in the across-track direction.

We apply the same transducer array echoes to compare the imaging performance that the effective TOAs and DOAs are estimated to thin the 3D image. The whole rectangular array is employed as a 2D system to achieve the MBES imaging. Beamforming is also taken in the along-track direction by the few elements with a large spacing of 2D MBES. Classical MBSAS takes the SAS processing in the along-track direction and CBF in the across-track direction through the moving of the transducer array. The method proposed in this paper takes the accelerated deconvolved beamforming in the across-track direction after the SAS processing. The imaging comparisons are shown in Figure 12 with a target projection on the bottom.

Figure 12a indicates the SAS imaging processing result with a multi-subarray receiving structure. Although the dual balls can be separated in the along-track direction owing to the constant resolution of SAS, the 2D image limits the detailed surveying. The actual sizes, locations, and depth information of the dual balls cannot be obtained in 3D space. Beamforming processing in the across-track direction is necessary to achieve the 3D acoustical full-scan surveying.

2D MBES introduces the beamforming in the along-track direction that the targets can be detected in the 3D space. However, the imaging resolution in the along-track direction is still limited by the footprint inevitably that the dual targets cannot be separated, as shown in Figure 12b. The imaging in the across-track direction extends that cannot indicate the actual target size, which is limited by the beamwidth and the detecting range. Fortunately, MBSAS theory achieves a constant resolution in the along-track direction that the dual balls can be separated effectively. The positions and the target size in the along-track direction match the preset parameters. Nevertheless, the imaging of MBSAS in Figure 12c employs the CBF method in the across-track direction that the resolution still cannot break through the MBES imaging theory limits. The imaging extends inevitably, and the size of the targets distorts that need improving.

**Figure 13:**
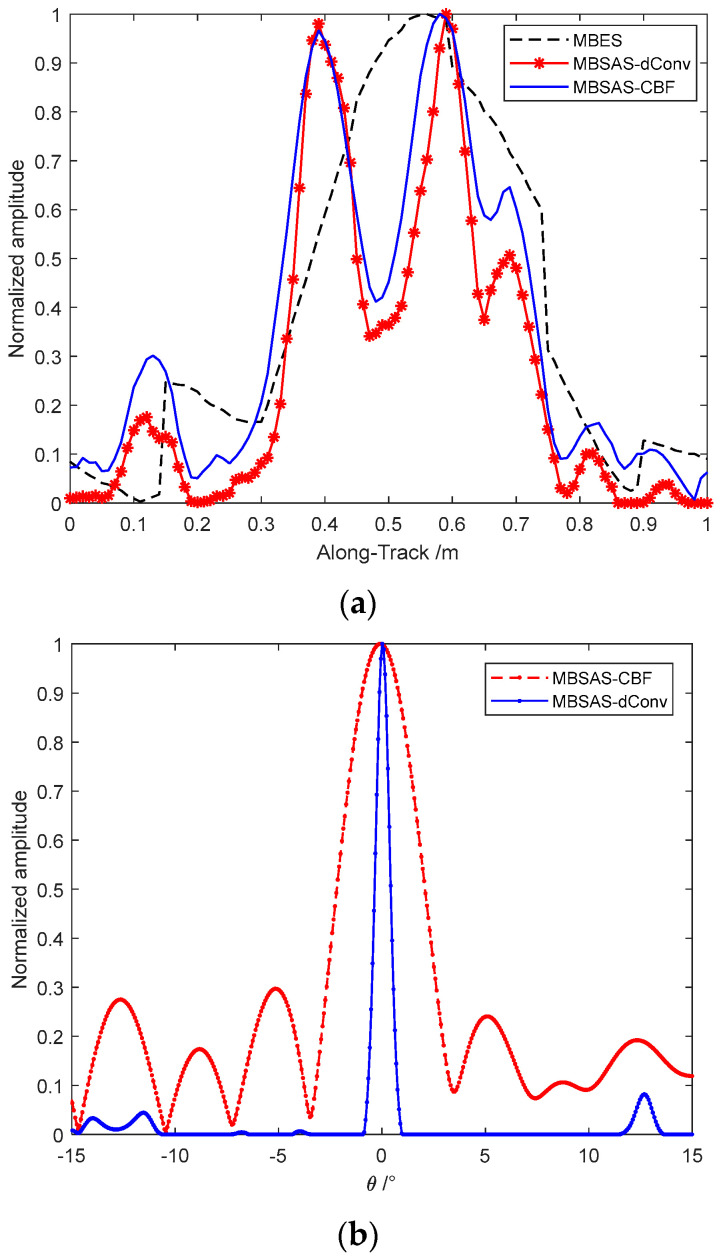
Sliced comparisons of different imaging methods: (**a**) sliced beamforming in the along-track direction of MBES, MBSAS with CBF, and MBSAS with deconvolved beamforming; and (**b**) sliced beamforming in the across-track direction through CBF and deconvolved beamforming of MBSAS.

The accelerated deconvolved beamforming employs the SAS pre-processed images that obtain dozens of complex images with a constant resolution in the along-track direction. Afterwards, iterations are taken based on the CBF output in Figure 12c and the PSF of the 2D transducer array. We obtain a high-resolution 3D image as shown in Figure 12d. The proposed method maintains the same constant resolution as SAS that also indicates the actual positions and size of the targets in the along-track direction. In the across-track direction, a narrow beamwidth helps to improve the imaging performance that the footprint of the target is obviously decreased. We also slice the image to observe the details and resolution in the across-track and along-track directions as Figure 13.

We sliced the beamforming result in the along-track direction to compare the resolution of MBES and the MBSAS which employs the CBF and accelerated deconvolved beamforming method in Figure 13a. The imaging of MBES in the along-track direction has serious aliasing that the dual targets cannot be separated owing to a wide beamwidth. MBSAS employs the SAS theory in the along-track direction that the constant resolution is an excellent benefit to the imaging system. MBSAS separates the dual balls with the correct targets’ locations. The SAS processing and beamforming processing can be separated into two individual steps. That means whether the imaging employs CBF or deconvolved beamforming, they have the same resolution in the along-track direction in theory. In other words, they have an almost identical beamwidth. Even though, the deconvolved beamforming method has a better ability to reduce the echo noise and decrease the sidelobe level than MBES.

We compare the CBF and deconvolved beamforming result, which is sliced shown in Figure 13b. The beamwidth of the CBF output is limited by the actual transducer array aperture that performs as $\Delta \theta =3.5^{\circ }$. Furthermore, the CBF has a high sidelobe level that we cannot distinguish between the weak target peak and the sidelobe. The accelerated deconvolved beamforming achieves a narrow beam as $\Delta \theta =1.0^{\circ }$ in 10 times iterations. The sidelobe level is also suppressed that the false peaks are cleared. Furthermore, the narrow beamwidth helps to improve the resolution in the across-track direction.

**Figure 14:**
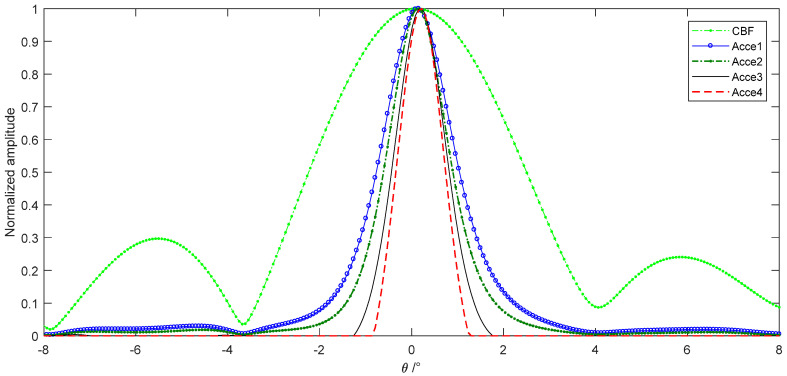
Imaging performance of different acceleration methods.

The convergence performance of different acceleration methods is also measured by the tank experiment above and sliced performed as Figure 14. Acceleration algorithm 1 is taken as the classical R-L of Equation (14) and its computation is improved through FFT. Acceleration algorithm 2 is taken by the exponential correction parameter p=1.5. Acceleration algorithm 3 is the imaging result of the vector extrapolation method. Acceleration algorithm 4 is the method we proposed in this paper that the vector extrapolation and the iterative function are both accelerated by the exponential parameter.

All the deconvolution algorithms are achieved through 10 times iterations, which is acceptable for engineering applications. Acceleration algorithm 1 has already achieved an appreciable improvement than CBF with a narrower beam. Acceleration algorithm 2 is taken by the exponential correction parameter that has a better convergence velocity. Acceleration algorithm 3 has a significant acceleration improvement than the exponential correction method. Acceleration algorithm 4 has a narrower beamwidth than acceleration algorithm 3, which means the better convergence velocity. Not only the beamwidth managed through the deconvolution methods, but also the sidelobe level has been improved obviously.

The tank experiment evaluates the imaging performance of MBSAS which employs the accelerated deconvolved beamforming method. The comparison indicates that the method proposed can effectively improve the beamwidth of the main lobe and suppress the sidelobe level, which is an excellent benefit to the detecting system. This technology can also be widely promoted in the field of underwater acoustic detection which employs finite elements.

## 6. Conclusions

In this paper, an accelerated deconvolved beamforming is proposed to improve the imaging performance of MBSAS system. The exponential acceleration and vector extrapolation method are combined to increase the convergence velocity of the classical R-L iterative algorithm. FFT is employed to increase the computation speed instead of the convolution and cross-correlation in the time domain. Single point target is simulated to compare the beamwidth and sidelobe level of different beamforming methods. The simulation indicates that the accelerated deconvolved beamforming proposed can effectively achieve a narrow beam with a low sidelobe level in several iterations. The feasibility of the method proposed is proved through the field experiment of MBES and tank experiment of MBSAS. The accelerated deconvolved beamforming can effectively improve the beamwidth and sidelobe level of the beamforming in the across-track direction, which is an excellent benefit to the underwater acoustic imaging system and can be widely promoted in the field of underwater acoustical remote sensing.

## Figures and Tables

**Table 1:** Solid target simulation parameters.

Parameters	Values	Parameters	Values
Echo frequency	150 kHz	Signal bandwidth	20 kHz
Elements on the across-track	32	Element spacing on the across-track	5 mm
Elements on the along-track	4	Element spacing on the along-track	110 mm
Transmitter aperture size	160 mm	Synthetized aperture	4 m
Cubical target size	2.0 m×0.8 m×2.0 m	Highlight spacing	100 mm

**Table 2:** Field experiment parameters of MBES.

Parameters	Values	Parameters	Values
Echo frequency	200 kHz	Waveform	CW
Beamwidth	1.0°	Pulse width	250 μs
Number of elements	100	Element spacing	3.75 mm
Swath width	160°	Depth	60 m

**Table 3:** Tank experiment parameters.

Parameters	Values	Parameters	Values
Echo frequency	150 kHz	Signal bandwidth	20 kHz
Elements on the across-track	32	Elements on the along-track	4
Transmitter aperture size	160 mm	Sampling positions	7
Interval on the along-track	15 cm	Synthetized aperture	1.05 m
